# *Rhabdias bufonis* (Rhabdiasidae) from the lung of the African common toad, *Amietophrynus regularis* (Bufonidae) in Egypt: new data on the basis of light and scanning electron microscopic study

**DOI:** 10.7717/peerj.5328

**Published:** 2018-07-20

**Authors:** Kareem Morsy, Sara Ali Mohamed, Fathy Abdel-Ghaffar, Hoda El-Fayoumi, Heba Abdel-Haleem

**Affiliations:** 1 Department of Biology, College of Science, King Khalid University, Abha, Saudi Arabia; 2 Department of Zoology, Faculty of Science, Cairo University, Cairo, Egypt; 3 Departement of Zoology, Faculty of Science, Beni Suef University, Beni Suef, Egypt

**Keywords:** *Rhabdias bufonis*, Nematoda, *Amietophrynus regularis*, Bufonidae, Cuticular elevations, Cephalic papillae, Morphology, Morphometry

## Abstract

**Background and Aims:**

*Rhabdias* sp. (Rhabdiasidae) is a nematode parasite of family Rhabdiasidae infecting the lung of amphibians. The present study provides new morphological details for *Rhabdias bufonis* isolated from the lungs of the African common toad, *Amietophrynus regularis* based on observations of light and scanning electron microscopy (SEM).

**Methods:**

Forty specimens were collected from its natural habitat: the damp, moist fields and gardens at Giza governorate, Egypt. Worms were isolated from the lungs, fixed and then preserved. They were examined using light and SEM with further line drawings.

**Results:**

Fourteen specimens (35%) were found to harbor *Rhabdias* with an intensity of three to five worms per host. Bodies of the gravid females were elongated, slender, measured 3.22–9.86 (5.64 ± 0.03) long and 0.09–0.48 (0.23 ± 0.02) wide at mid-length. Buccal capsule was with cylindrical lumen and sclerotized walls. Ovaries were almost straight. The uteri were located anterior and posterior to the vulva. Uterus were filled with a large number of eggs (17–42). Eggs were oval in shape and some of them were with fully developed larvae inside. The tail was comparatively short, gradually tapered. SEM showed that worms possessed an oval anterior end with a simple, slit like oral opening. The lipless edges of the mouth opening were bordered with tiny cuticular elevations, radiated outwards. Two pairs of submedian cephalic papillae were observed around the oral opening as well as two amphids.

**Conclusion:**

The current study presents new morphological details for *R. bufonis* isolated from the African common toad. Also, the morphology of the slit-like mouth opening, the two pairs of cephalic papillae, two amphids and the three pairs of cuticular elevations supporting the area around mouth opening were investigated.

## Introduction

Studies on the helminth fauna of amphibians have received much attention in the recent years (Dusen & Oguz, 2010; Akani et al., 2011; Santos, Amato & Márcio Borges 2013). The recent interest in amphibian parasites stems from the declining in amphibian populations (Johnson et al., 2001; Ibrahim, 2008). Nematodes of the genus *Rhabdias* Stiles et Hassall (1905) are a large group of lung-dwelling parasites and comprises about 80 nominal species of the nematodes parasitic in amphibians and reptiles (Kuzmin & Tkach, 2017). Morphologically they are females; however, hermaphroditism has been reported for some species of the genus, based on testis zones and sperm cells in the ovaries (Runey, Runey & Lauter, 1978). The life cycles of these nematodes alternate between parasitic and dioecious free-living generations. Parasitic species of *Rhabdias* are infecting the lungs of amphibians and reptiles (Anderson, 2000; Kuzmin, 2013; Tkach, Kuzmin & Snyder, 2014). Of about 39 species of *Rhabdias* parasitizing anuran hosts, eight species of four families have been reported from Afrotropical region (Junker et al., 2010). Twenty four species of *Rhabdias* are known from Africa (Kuzmin & Tkach, 2017). Of them, 13 are parasitic in Chamaeleonidae lizards while 11 are parasites of amphibians. In Egypt, three different *Rhabdias* species were reported, *Rhabdias bufonis* Schrank (1788) (Moravec, Baruš & Ryšavý, 1987), *R. aegyptiaca* El–Garhy & Garo (2006) and *Rhabdias* sp. (left unnamed) from the lungs of maculated toad *Amietophrynus regularis* at Aswan governorate (Saad, Khalifa & Mostafa, 2009). Due to their similarity in the morphological characteristics such as the labial structures, the position of vulva, the tail shape and absence of males in parasitic generations, the differentiation between species belonging to the genus *Rhabdias* became complicated (Tkach, Kuzmin & Pulis, 2006; Kuzmin, Tkach & Brooks, 2007; Saad, Khalifa & Mostafa, 2009). However, the morphology of the anterior end of some *Rhabdias* species yields some characters appropriate for the species diagnosis (Kuzmin, Tkach & Vaughan, 2005). Tkach, Kuzmin & Pulis (2006), Kuzmin, Tkach & Brooks (2007) and Martínez-Salazar, Pérez-Ponce de León & Parra Olea (2009) suggested that additional taxonomical tools like molecular biology techniques, scanning electron microscopy (SEM), host specificity and geographic distribution are required to diagnose species of this genus. The African common toad, *A. regularis* Reuss (1833) is widespread in the Sub-Saharan Africa, with its range extending to the oases in Algeria and Libya as well as to northern Nilotic Egypt (Frost, 2014). According to Rödel (2000) and Ibrahim (2008) the prey of *A. regularis* often includes beetles, ants, bugs, insects, grubs, slugs, worms and other invertebrates. The present study provides new morphological details for the nematode *R. bufonis* isolated from the lungs of the African common toad, *A. regularis* by light and SEM.

## Materials and Methods

Forty specimens of the African common toad, *A. regularis* were collected by hand or noose from its natural habitat: the damp, moist fields and gardens at Giza governorate, Egypt (30°2′N and 31°12′E) from March to August 2017. Animals were subjected to euthanasia using 20% benzocaine gel (Anbesol, Pfizer, Inc., New York, NY, USA). Each specimen was subsequently necropsied and all organs were examined searching for helminthes using a ZEISS Compact Greenough stereomicroscope (Model Stemi 305; Zeiss, Oberkochen, Germany). Where necessary, all animal procedures were carried out according to the regulatory laws regarding experimental Animal Ethics Committee, Faculty of Science, Beni-Suef University, Beni-Suef, Egypt (Ethical Approval Number: 2015/10). Nematode worms were isolated from the lungs, heat fixed in 10% neutral buffered formalin for 15 min and then preserved in 70% ethanol in 5% glycerol solution to avoid sudden drying. Finally, samples were transferred to lactophenol for clearance. The prepared samples were examined using differential interference contrast light microscopy with digital image analysis system (analysis auto 5.0). Drawings were made with the aid of a camera lucida. Measurements were in millimeters unless otherwise stated. For SEM, samples were fixed in 4% glutaraldehyde in 0.1 M sodium cacodylate buffer, washed in the same buffer, and dehydrated in a graded alcohol series (50%, 60%, 70%, 80%, 90% and 100%). Samples were then processed in a critical point drier “Bomer–900” with freon 13, sputter-coated with gold–palladium in a Technics Hummer V, and finally examined with a Jeol scanning electron microscope (Model JSM7610F; Jeol, Tokyo, Japan).

## Results

### Morphology of *R. bufonis* (Schrank, 1788)

Description is based on 28 gravid adults; morphometric data is presented as a range followed by the mean ± SD in parentheses.

#### Light microscopy

**Figure 1:**
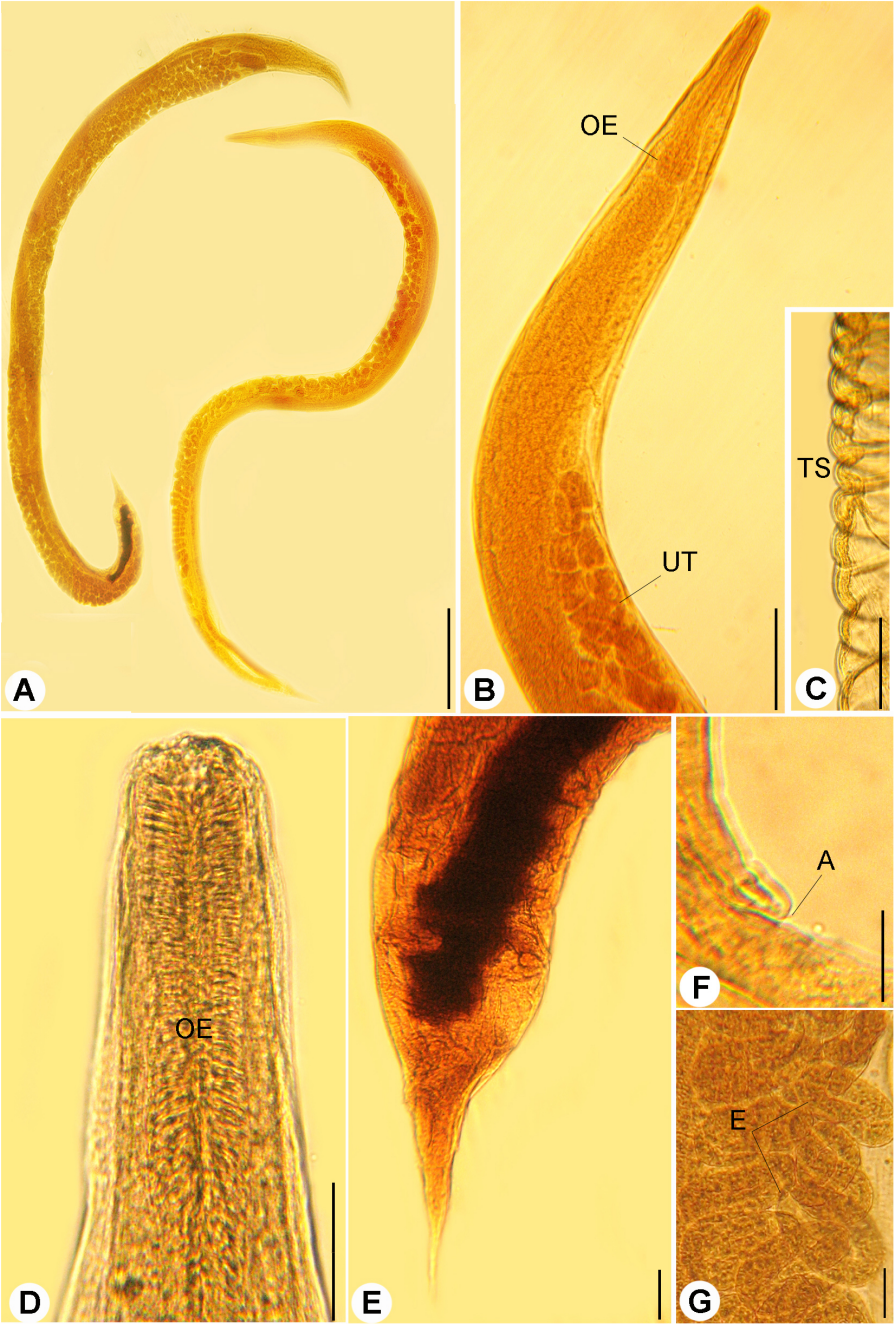
Light micrographs of *R. bufonis*. (A) Females, left and right lateral views, scale bar 0.7 mm. (B) Anterior part, lateral view; OE, esophagus; UT, uterus, scale bar 0.17 mm. (C) Transverse striations (TS) of cuticle, scale bar 0.03 mm. (D) Magnified anterior end, scale bar, 0.03 mm. (E) Tail region, scale bar 0.07 mm. (F) Anus (A), scale bar 0.06 mm. (G) Eggs (E), scale bar 0.04 mm.

**Figure 2:**
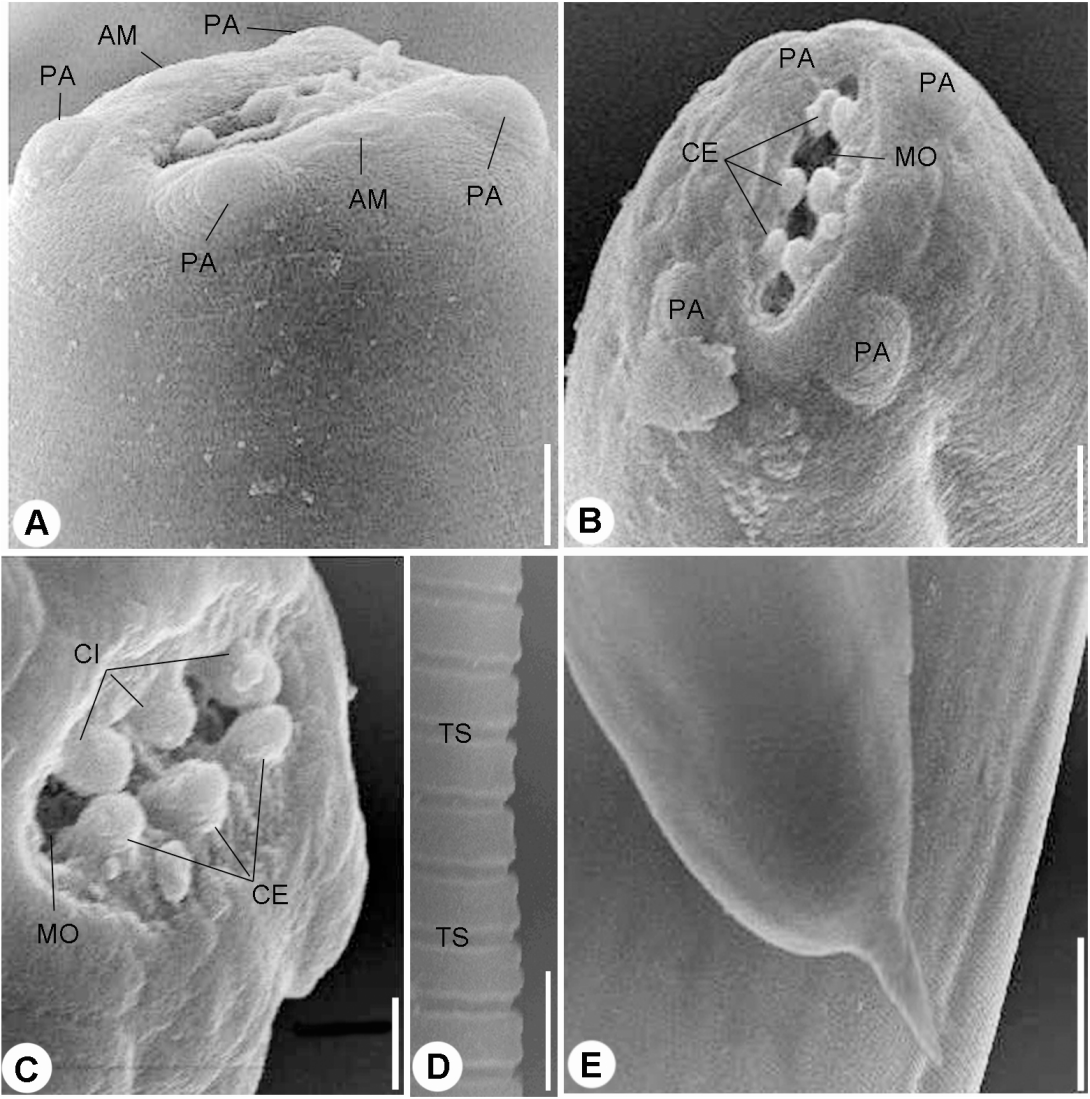
Scanning electron micrographs. (A) and (B) Cephalic end, lateral (A) and Apical (B) views, four papillae (PA) and two amphids (AM) surrounding mouth opening (MO), scale bars 10 μm. (C) Magnified apical view for the cuticular inflation (CI), scale bars 5 μm. (D) Transverse striations (TS) of cuticle, scale bar 10 μm. (E) Tail end, scale bar 0.50 μm.

Bodies of the gravid females were elongated, slender (Figs. 1A and 1B) measured 3.22–9.86 (5.64 ± 0.03) long and 0.09–0.48 (0.23 ± 0.02) wide at mid-length. Cuticle inflation and transverse striations (Fig. 1C) were observed, inflation widened from anterior end to level of esophagus then rapidly narrowed towards caudal region with pronounced inflation. Buccal capsule was with cylindrical lumen and sclerotized walls (Figs. 2B and 2C), 13–31 (18 ± 2) μm long and 16–27 (24 ± 2) μm wide. The anterior part of esophagus contacting posterior end of buccal capsule, esophagus was muscular, 0.27–0.63 (0.18 ± 0.02) long and 0.25–0.5 (0.30 ± 0.03) as a maximum width with muscular anterior third and elongated posterior bulb (Figs. 1B and 1D). Nerve ring surrounding esophagus and measured 0.168–0.240 from the anterior end. Anterior end of intestine connected to the esophageal bulb, it was widened at the esophageo-intestinal junction. The content of the intestine was black at their posterior part. Reproductive system was amphidephlic. Ovaries were almost straight. The uteri were located anterior and posterior to the vulva. Vulva was nearly equatorial 1.47–5.98 from the anterior end. Uterus filled with eggs, 17–42 in number. Eggs were oval in shape (Fig. 1G), 0.120–0.132 (0.126 ± 0.002) × 0.039–0.081 (0.051 ± 0.02) in size and some of them were with fully developed larvae inside. The tail was comparatively short, gradually tapered, tail length 0.131–0.435 (0.320 ± 0.02) long (Fig. 1E).

#### Scanning electron microscopy

Scanning electron microscopy showed that the nematode isolated in the present study possessed an oval anterior end (Figs. 2A and 2B) with a simple, slit like oral opening. The lipless edges of the mouth opening were bordered with tiny cuticular elevations, radiating outwards (Figs. 2B and 2C).

Two pairs of submedian cephalic papillae (two dorso-lateral and two ventro-lateral) were observed around the oral opening as well as two amphids (Figs. 2A and 2B). The transverse cuticle striations (Fig. 2D) and the pointed tail (Fig. 2E) were prominent by SEM.

**Figure 3:**
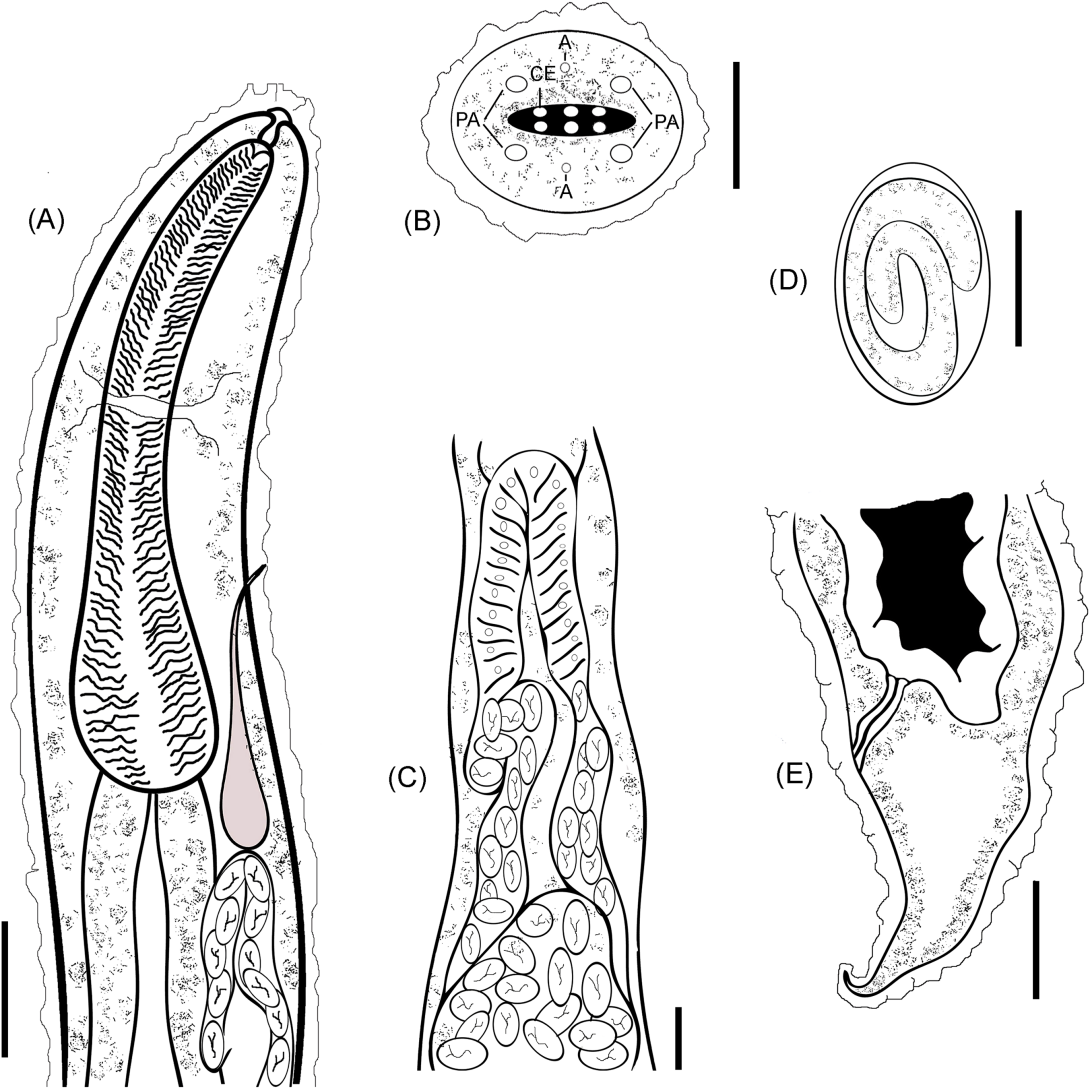
*R. bufonis*, parasite of *A. regularis*. (A) Anterior part, lateral view, scale bar 0.16 mm. (B) Cephalic end, lateral view, scale bar 20 μm. (C) Loop of anterior genital tube, scale bar 0.14 mm. (D) Egg, lateral view, scale bar 0.07 mm. (E) Tail end, lateral view, scale bar 0.32 mm. PA, Cephalic papillae; CI, Cuticular inflation; AM, Amphids.

A line drawing showing a diagrammatic representation for the anterior part (Fig. 3A), cephalic end (Fig. 3B) and cuticular elevations (Fig. 3C), loop of anterior genital tube (Fig. 3D), and the tail end (Fig. 3E) of *R. bufonis* was illustrated.

## Taxonomic Summary

*Species*: *Rhabdias bufonis* (Schrank, 1788).

*Host*: *Amietophrynus regularis* Reuss, 1833 (Family: Bufonidae).

*Site of infection*: Lungs.

*Locality*: Giza governorate, Egypt.

*Prevalence and intensity of infection:* Fourteen out of 40 (35%) hosts were infected with an intensity of three to five (4 ± 1) worms per one host.

*Deposition*: Permanent slides as well as 70% preserved gravid females were deposited at the Parasitology division, Zoology department museum, Faculty of Science, Cairo University, Egypt with accession number (C/Para/11/2018).

## Discussion

The differentiation among species belonging to the genus *Rhabdias* is often complicated due to their high morphological uniformity (Chu, 1936; Baker, 1978; Tkach, Kuzmin & Pulis, 2006; Kuzmin, Tkach & Brooks, 2007). Within the species of *Rhabdias*, the shape of cephalic end represents an important character for the differentiation between species and genera of the family Rhabdiasidae (Railliet, 1915) and yields characters suitable for species diagnostics (Kuzmin, Tkach & Vaughan, 2005). Genus *Rhabdias* can be divided into three groups based on the morphology of their head end according to Baker (1978): species without lips, with six lips and with two lateral pseudolabia. Three different *Rhabdias* species were reported from amphibian hosts belonging to family Bufonidae, represented by a single genus, *Amietophrynus* (formerly included in *Bufo*), in the Afrotropical region. These were *R. bufonis* Moravec, Baruš & Ryšavý (1987), *R. picardiae* Junker et al. (2010) and *R. africanus* Kuzmin (2001). By comparing the recovered parasite with different species of the same genus previously recorded in the Afrotropical region, it was found that the present species is morphometrically similar to *R. bufonis* described previously from the same host by Moravec, Baruš & Ryšavý (1987) while many characters are different to those of the comparable species. Both species resemble each other by possessing similar head structure (absence of lips, presence of small submedian mouth papillae); the intestinal apex is broader than the esophageal base and the equatorial position of vulva. *R. bufonis* recorded in the present study has a longer body (9.86 vs. 8.35 mm), a shorter esophagus (270–630 vs. 690–790) and a longer buccal capsule (13–41 vs. 8–10 mm) than *R. picardiae* and differ from *R. africanus* in the body size and the absence of two lateral pseudolabia. The present form, differs from the description of *R. bufonis* given by Travassos (1930) and Hartwich (1972) in the body length (3.22–9.86 vs. 9–12) and in the size of buccal capsule which is of diagnostic importance (Lhermitte-Vallarino et al., 2008; Junker et al., 2010). The current study introduced new morphological characteristics for the nematode *R. bufonis* isolated from the lung of *A. regularis* based on SEM. One of the most important features of *R. bufonis* is the cephalic end structure which has been overlooked by previous studies. In Egypt, only three different species of the genus *Rhabdias* have been described based on the light microscopic studies and these species are: *R. bufonis* Moravec, Baruš & Ryšavý (1987), *R. aegyptiaca* El–Garhy & Garo (2006) and *Rhabdias* sp. Saad, Khalifa & Mostafa (2009). The parasite recovered in the present study differs from *R. aegyptiaca* where the later possesses six lips with a unique arrangement around the mouth opening, cuticle lined pores on its lateral surface and the size of the buccal capsule which is of diagnostic importance (Lhermitte-Vallarino et al., 2008; Junker et al., 2010). Also, it differs from *Rhabdias* sp. reported by Saad, Khalifa & Mostafa (2009), where it has four developed lips and two lateral teeth on the mouth opening, the cuticular swelling that covers the whole body and the post-equatorial position of vulva. *Rhabdias* species that were recovered from a bufonid member with a sub-Saharan distribution in Egypt were compared in Table 1.

**Table 1:** *Rhabdias bufonis* (female, present study) and members of the same genus previously reported in Egypt.*

Species	Length	Width	Buccal capsule	Teeth	Esophagus	Vulva	Tail length	Eggs	Reference
***R. bufonis***	2.99–13.02	0.136–0.476	0.015	Absent	0.288–0.510	Equatorial	0.144–0.420	L: 0.117–0.144 W: 0.051–0.72	Moravec, Baruš & Ryšavý (1987)
***R. aegyptiaca***	8–10	0.3–0.5	–	Absent	550 μm	Equatorial	170–200 μm	66 μm in length	El–Garhy & Garo (2006)
***Rhabdias* sp**	5.2–12.5	0.2–0.7	0.01–0.032	Two lateral teeth	0.25–0.5 (0.3)	Post-equatorial	0.23–0.4 (0.3)	L: 0.1–0.12 W: 0.06–0.08	Saad, Khalifa & Mostafa (2009)
***R. bufoni***	3.22–9.86 (5.64 ± 0.03)	0.09–0.48 (0.23 ± 0.02)	L: 13–31 (18 ± 2) μm W:16–27 (24 ± 2) μm	Absent	L: 0.27–0.63 (0.18 ± 0.02) W: 0.25–0.5 (0.30 ± 0.03)	Equatorial	0.131–0.435 (0.320 ± 0.02)	L: 0.120–0.132 (0.126 ± 0.002) W: 0.039–0.081 (0.051 ± 0.02)	Present study

**Note:**

* Measurements in mm, otherwise stated.

## Conclusion

The current study presents new morphological details for *R. bufonis* isolated from the African common toad. Also, the morphology of the slit-like mouth opening, the two pairs of cephalic papillae, two amphids and the three pairs of cuticular elevations supporting the area around mouth opening were investigated. This is the second report for this parasite in Egypt representing an important reference for upcoming studies.

## Additional Information and Declarations

### Data Availability

The following information was supplied regarding data availability: The specimens and slides for the parasite described in this article are stored at the Zoology department, Faculty of Science, Cairo University, Egypt (accession: C/Para/11/2018).
